# DQ2, DQ7 and DQ8 Distribution and Clinical Manifestations in Celiac Cases and Their First-Degree Relatives

**DOI:** 10.3390/nu7064955

**Published:** 2015-06-18

**Authors:** Magdalena Araya, Amaya Oyarzun, Yalda Lucero, Nelly Espinosa, Francisco Pérez-Bravo

**Affiliations:** 1 Human Nutrition, Institute of Nutrition and Food Technology (INTA), University of Chile, Santiago, Chile; E-Mail: amaya.oyarzun@gmail.com; 2 Department of Pediatrics, Faculty of Medicine, University of Chile, Santiago, Chile; E-Mail: ylucero@gmail.com; 3 Hospital Militar, Santiago, Chile; E-Mail: nespinosap@yahoo.es; 4 Nutrition Department, Faculty of Medicine, University of Chile, Santiago, Chile; E-Mail: fperez@med.uchile.cl

**Keywords:** celiac disease, first degree relatives, HLA- DQ2, HLA-DQ7, HLA-DQ8

## Abstract

HLA-linked genes are relevant to celiac disease (CD); the potential genetic differences present worldwide are not fully understood. Previous results suggest that the distribution of HLA-DQ2/DQ7/DQ8 in Chile may differ from that in Europe and North America. In celiac patients and their first-degree relatives (FDRS), we assessed their clinical, serological and histological characteristics, determined HLA-DQ2, HLA-DQ7 and HLA-DQ8 alleles and genotypes, and evaluated the relations between them. A total of 222 individuals were assessed (56 cases, 166 FDRs). 16.9% of FDRs were tTG positive; 53.6% of them showed overweight/obesity and 3% undernourishment; they spontaneously declared being asymptomatic, but detailed questioning revealed that 60.7% experienced symptoms, which had not been investigated. DQ2 was present in 53.9% and 43.9.0% of cases and FDRs (*p* < 0.05). The most frequent genotype distribution was DQ2/DQ7 (fr 0.392 (cases) and 0.248 (FDRs), respectively, *p* < 0.02). The next most common genotypes were HLA-DQ2/DQ8 (fr 0.236 in FDRs and 0.176 in cases, *p* < 0.05). 3.92% cases were not HLA-DQ2/DQ8 carriers. Among tTG positive FDRs, 57.4%, 22.3% and 20.2% carried DQ2, DQ7 and DQ8, respectively. In cases, 72.7% of the biopsies classified Marsh ≥3 carried at least one DQ2; 91.7% of DQ2/DQ2 and 88.3% of DQ2/DQ7 were Marsh ≥3. Thus, DQ2 frequency is lower than reported; the higher frequency found for DQ8 and DQ7 concur with recent publications from Argentine and Brazil. These results suggest that although CD may manifest clinically in ways similar to those described in other populations, some genetic peculiarities in this region deserve further study.

## 1. Introduction

Celiac disease (CD) is a heritable, frequent, chronic disorder that involves small intestinal inflammation and autoimmune manifestations in response to dietary gluten. The condition results from the interplay of genetic and environmental factors (mainly dietary gluten) [1,2,3]; the host immune system and microbiota are also involved in the development of CD [2,4,5,6,7]. Although knowledge has progressed greatly in the last decades, several aspects of the condition remain unclear. Until the 90s, CD was thought to occur mainly among white Europeans [8,9] and there was no clear explanation as to why countries with rather large white populations of European origin reported low incidence of the disease [10]. The significant geographical differences reported [9] refer mainly to prevalence of CD [11,12,13] while genetic characteristics in some areas of the globe are still insufficient and not fully understood. It is well-agreed that a significant proportion of the genetic predisposition for CD comes from human leukocyte antigen (HLA)-linked genes, estimated to account for up to 40% of the genetic load [14]. The southern cone of South America is one of the areas where available evidence is incomplete and still unclear. The estimated prevalence of CD in Chile [15] and in Argentine [16] is 0.6%; in both countries and HLA-DQ8 frequencies seem to be higher than described elsewhere [17,18]. Also, there is data suggesting that the frequency of cases not carrying HLA-DQ2 or DQ8 may be higher than reported in other areas [19,20]. It has been speculated that these features might be related to common native genetic (Mapuche) background; in Chile, there is evidence that diabetes mellitus (DMT1) would be infrequent in the Mapuche native group [21]. The first objective in this protocol was to assess the frequency and distribution of HLA-DQ2, HLA-DQ8 and HLA-DQ7 in a family study of celiac patients and their first-degree relatives (FDRs). 

Better diagnostic tools and recognition of the extra intestinal and autoimmune manifestations have proved that CD is frequent in most countries that actively search for it. Recognition of atypical, incomplete or non-classical forms of CD and the application of active search in risk groups has yielded good evidence for an increased prevalence of CD among FDRs of celiac patients [17,22,23,24,25,26]. However, evidence about the global frequency of the disease in this group and their clinical and genetic characteristics is largely insufficient. In an effort to contribute to expand our knowledge on these issues, we set as a second objective the determination of serologically positive FDRs of known celiac cases (susceptible group) and their clinical characterization.

Thus, in this study we measured HLA-DQ2, HLA-DQ8 and HLA-DQ7 in celiac cases and their FDRs and characterized their symptoms, nutritional status, serological and histologic data. Then we calculated the percentage of serologically positive FDRs, analyzed their characteristics and compared serologically positive FDRs with cases and serologically negative FDRs. Finally, we related the genetic findings to clinical and serological characteristics of cases and FDRs.

## 2. Experimental Section

### 2.1. Patients and First-Degree Relatives (FDRs)

Patients consulting for CD at the outpatient clinic of INTA during 2012–2014 were evaluated as potential candidates for the study. Following current diagnostic criteria [3,22,27] the 57 biopsy-proven celiac patients and their 171 FDRs were invited to participate in the study. Participants gave their written informed consent prior to incorporation to the protocol. INTA’s Institutional Review Board approved the study. Cases and their families participated in an interview where the medical history of each family member was registered, including previous consultations and diagnoses; the presence of clinical symptoms was asked for in the form of “do you have it”, “did you have it at the time of diagnosis” and “have you ever had it”.

Clinical presentations were defined on the basis of symptoms present at diagnosis—classical when initial diagnosis was led to by digestive symptoms, non-classical when non-digestive symptoms (extra-intestinal and/or autoimmune manifestations and/or being a FDR) led to the initial investigation. At the end of the interview, weight and height were registered and nutritional status was classified following the World Health Organization (body mass index (BMI) for adolescents and adults and Z score weight/height for children under 5 years) [28]. A blood sample was obtained from the antecubital vein for serological and genetic analyses. 

### 2.2. Laboratory testing

Serum IgA was measured using an ELISA commercial kit (Alpco^®^, Salem, NH, USA). IgA-anti-endomysial antibodies (EMA) were determined by indirect immunofluorescence using slides with monkey esophagus sections as substrate (IMMCO Diagnostics^®^, Buffalo, NY. USA), IgA anti-tTG2 antibodies (tTG) were measured using a commercial ELISA kit (AESKU^®^, Wendelsheim, Germany), expressing the results as suggested by the manufacturer: negative ≤ 12 U, borderline = 12–18 U and positive ≥ 18 U. When IgA-tTG was negative and total serum IgA was below the cut off for age, IgA/IgG tTG was measured using Celicheck (a kit that measures IgA- and IgG- transglutaminase antibodies, (AESKU^®^, Wendelsheim, Germany), expressing the results as negative ≤ 16 U, borderline = 16–24 U and positive ≥ 24 U. Mucosal lesions in the small intestinal biopsies were graded according to Marsh classification [29]: M1 = more than 25 IELs/100 enterocytes in the epithelium (lymphocytic enteritis); M2 = with crypt hyperplasia; M3a = with moderate villous atrophy, M3b = with subtotal villous atrophy; M3c = total villous atrophy. Genetic studies were performed using the commercial kit DQ-CD Typing plus (BioDiagene^®^, Palermo, Italy). After blood lysis, DNA was amplified by polymerase chain reaction (PCR) (12 per sample) and identification of alleles was carried out by agarose gel electrophoresis. HLA- DQ typing for celiac susceptibility included the single sequence specific primer-PCR home based method to detect the presence of the HLA heterodimer DQA1*0501 - DQB1*0201 [30,31]. Then we used a sequence specific oligonucleotide-PCR based method to type the HLA DQB1*locus (Dynal Biotech Ltd, Bromborough, UK) to confirm the presence/absence of the DQB1*02 allele or to verify the presence of the other DQB1*03 risk allele. Finally, the sequence-specific primer-PCR technique (Dynal Biotech Ltd, Bromborough, UK) was used to resolve the DQB1*03 locus, showing the presence/absence of the DQB1*0302 allele. HLA DR typing HLA-DRB1 typing was performed by a sequence specific oligonucleotide-PCR-based method (Dynal Biotech LTD, Bromborough, UK) that determined the phased DR-DQ genotypes of all individuals. Results are expressed nominating DQ2 = DQA1*0501, DQB1*0201. DQ7 = DQB1*0301/04. DQ8 = DQA1*03, DQB1*0302.

### 2.3. Statistical Analysis

Analysis of general data included descriptive statistics for variables with normal or non-normal distribution. Allele and genotype frequencies were computed as sample proportions. Comparison of such frequencies in cases and FDRs was statistically assessed by a chi-square test corrected by multiple comparisons [26].

## 3. Results 

### 3.1. Clinical Data

A total of 56 out of 57 celiac cases agreed to participate, providing 166 FDRs (48 fathers, 50 mothers, 8 sons/daughters and 60 siblings). Their sex distribution, mean age and nutritional status are shown in Table 1. Women were more frequent among patients with classical presentations and in symptomatic FDRs. Only three (non-classical) cases were under 8 years of age at diagnosis; they were investigated because of anemia (*n* = 1) or failure to thrive (*n* = 2). Overweight/obesity was present in 21.4% and 53.6% of cases and FDRs, respectively. There was no morbid obesity in cases or FDRs; 10.7% of cases and 3% of FDRs were undernourished.

**Table 1:** Sex, age and nutritional status in celiac cases and their first degree relatives.

	Cases (*n* = 56)		First Degree Relatives (*n* = 166)		Total (*n* = 222)
	Classical	Non-Classical	Symptomatics	Subclinical	
**Female**s *n* (%)	32 (68.1)	4 (44.4)	57 (66.3)	33 (41.3)	126 (100)
**Age** y (mean)					
0–8	19 (4.2 y)	3 (4.5 y)	9 (4.5 y)	8 (4.0 y)	39 (100)
8–12	5 (9.7 y)	4 (9.4 y)	2 (10.1 y)	7 (10.2 y)	18 (100)
12–18	6 (14.9 y)	2 (12.7 y)	7 (14.3 y)	4 (14.6 y)	19 (100)
>18	17 (31.1 y)	0	68 (42.9 y)	61 (44.1 y)	146 (100)
**Nutritional status**					
Undernourished	4 (8.5)	2 (22.2)	3 (3.5)	2 (2.5)	11 (100)
Well nourished	31 (66)	7 (77.8)	39 (45.3)	33 (41.3)	110 (100)
Overweight	11 (23.4)	0	29 (33.7)	33 (41.3)	73 (100)
Obesity type I	1 (2.1)	0	12 (14)	11 (13.8)	24 (100)
Obesity type II	0	0	3 (3.5)	1 (1.3)	4 (100)
Total	47 (100)	9 (100)	86 (100)	80 (100)	222

Twenty cases (35.7%) initially presenting classical digestive features required hospitalization at diagnosis. Among celiac cases, both in those initially investigated for classical digestive symptoms or other reasons, abdominal distension, abdominal pain, weight loss/undernutrition and diarrhea were the most frequent findings (Table 2). Many FDRs declared they were asymptomatic, but detailed and directed questioning revealed that they experienced a variety of symptoms (Table 2), abdominal pain and constipation being the most frequent ones. In 23.2% of cases and in 12% of FDRs an autoimmune condition was already diagnosed at the time of this study, Hashimoto’s thyroiditis being the most frequent diagnosis. Since appearance of symptoms, children and adult patients’ first consultation occurred after 0.1 and 5.9 years and diagnosis was reached after 0.9 and 6 years, respectively. No differences were detected between genders in time to first consultation or to reach diagnosis. Cases with non-classical courses consulted and reached diagnosis in shorter times in comparison to patients with classical symptoms; comparing the periods 2000–2009 and 2010–2014, mean time to reach diagnosis among adult patients decreased from 108 months to 62 months.

**Table 2:** Symptoms, autoimmune diseases and other diagnoses detected in celiac cases and their first-degree relatives.

	Cases (*n* = 56)			First Degree Relatives (*n* = 166)		
	Total *n* (%)	Classical *n* (%)	Non-Classical *n* (%)	Total *n* (%)	Symptomatic *n* (%)	Subclinical *n* (%)
**Symptoms**						
Abdominal distension	49 (87.5)	44 (78.6)	5 (8.9)	2 (1.2)	2 (1.2)	0 -
Abdominal pain	41 (73.2)	37 (66.1)	4 (7.1)	48 (28.9)	48 (28.9)	0 -
Weight loss	41 (73.2)	36 (64.3)	5 (8.9)	1 (0.6)	1 (0.6)	0 -
Diarrhea	40 (71.4)	39 (69.6)	1 (1.8)	3 (1.8)	3 (1.8)	0 -
Malaise	33 (58.9)	32 (57.1)	1 (1.8)	1 (0.6)	1 (0.6)	0 -
Constipation	27 (48.2)	24 (42.9)	3 (5.4)	30 (18.1)	30 (18.1)	0 -
Steatorrhea	22 (39.3)	22 (39.3)	0 -	2 (1.2)	2 (1.2)	0 -
Vomiting	23 (41.1)	21 (37.5)	2 (3.6)	0 -	0 -	0 -
Malnutrition	20 (35.7)	16 (28.6)	4 (7.1)	2 (1.2)	2 (1.2)	0 -
Anorexia	14 (25.0)	13 (23.2)	1 (1.8)	0 -	0 -	0 -
Edema	8 (14.3)	8 (14.3)	0 -	1 (0.6)	1 (0.6)	0 -
**Autoimmune disease**						
Autoimmune thyroiditis	9 (16.1)	9 (16.1)	0 (0)	17 (10.2)	13 (7.8)	4 (2.4)
DM type 1	2 (3.6)	1 (1.8)	1 (1.8)	2 (1.2)	2 (1.2)	0 -
Down Syndrome	1 (1.8)	1 (1.8)	0 -	0 -	0 -	0 -
Autoimmune hepatitis	1 (1.8)	1 (1.8)	0 -	1 (0.6)	1 (0.6)	0 -

About half of cases showed positive serologic studies at the time of current assessment; in only 5 out of 25 tTG positive cases, EMA was also positive. Among FDRs, 28 out of 166 (16.9%) were tTG positive, of which 5 were also EMA positive (Table 3). All 28 positive FDRs were advised a small intestinal biopsy but only 10 (35.7%) underwent the procedure; of these 4 (2 adult women and 2 adult men) were diagnosed CD; two were eutrophic and two overweight, three had digestive symptoms and one was asymptomatic. In all, diagnosis was reached after the intestinal biopsy showed lesions Marsh ≥ 2 and clinical improvement after gluten free diet was demonstrated.

Biopsy records of cases were available in 44 of 56 cases (Table 4). Of these, 35 of 44 (79.5%) showed mucosal lesions classified as Marsh 3 or more; no differences were observed in the proportion of Marsh 3 lesions between classical and non-classical cases.

**Table 3:** Antiendomysial antibodies and antitransglutaminases in celiac cases and first-degree relatives.

	Cases (*n* = 56)		First Degree Relatives (*n* = 166)	
	Classical *n* = 47	Non-Classical *n* = 9	Symptomatic *n* = 83	Subclinical *n* = 84
EMA * (+)	5 (10.6%)	0 -	3 (3.6%)	2 (2.4%)
tTG ** (+)	25 (53.2%)	4 (44.4%)	19 (22.9%)	9 (10.7%)

* = Antiendomysial antibodies. ** = transglutaminase antibodies.

**Table 4:** Small intestinal biopsy lesions and genotypes in 44 cases of celiac disease.

		Genotypes							
		DQ2/DQ2	DQ2/DQ7	DQ2/DQ8	DQ7/DQ7	DQ7/DQ8	DQ8/DQ8	DQ8/ND	Total
**Marsh**	*n* (%)								
2–3	9 (20.5)	1 (2.3)	2 (4.5)	2 (4.5)	1 (2.3)	0	3 (6.8)	0	9 (100)
≥3	35 (79.5)	11 (25)	15 (34.1)	6 (13.6)	1 (2.3)	0	1 (2.3)	1 (2.3)	35 (100)
**Total**	44 (100)	12 (27.3)	17 (38.6)	8 (18.2)	2 (4.5)	0	4 (9.1)	1 (2.3)	44 (100)

ND = Not Detected (represents an allele not detected by the techniques used).

### 3.2. Genetic Data

Distribution of genotypes found and biopsy lesions observed in cases also appear in Table 4. In relation to duodenal lesion and DQ assessment, 91.4% of biopsies classified Marsh ≥ 3 carried at least one DQ2; 91.7% of DQ2/DQ2 was Marsh ≥ 3 and 88.2% were DQ2/DQ7 (Table 4). 

Table 5 shows the DQ2, DQ7, DQ8 allele and genotypic distribution in cases and FDRs. Contribution of DQ2 in celiac cases and FDRs was 53.9% and 43.9% (*p* < 0.05), respectively, with no differences detected for DQ7 or DQ8 distribution between CD cases and FDRs. 

**Table 5:** Alleles and genotype frequencies in 51 celiac cases and 157 tTG first-degree relatives (FDRs).

	Cases (*n* = 51) N (fr)	FDRs (*n* = 157) N (fr)	*p* value
**Alleles**			
DQ2	55 0.539	138 0.439	<0.05
DQ7	24 0.235	82 0.261	NS
DQ8	22 0.215	82 0.261	NS
Others	1 0.011	12 0.039	-
**Genotypes**			
DQ2/DQ2	13 0.255	27 0.172	NS
DQ2/DQ7	20 0.392	39 0.248	<0.02
DQ2/DQ8	9 0.176	37 0.236	NS
DQ2/ND	0	7 0.045	-
DQ7/DQ7	2 0.039	14 0.089	NS
DQ7/DQ8	0 0.000	15 0.096	-
DQ8/DQ8	6 0.118	14 0.089	NS
DQ8/DQ2	0	1 0.006	-
DQ8/ND	1 0.020	1 0.006	-
ND/ND	0	2 0.013	-

ND = allele not detected.

Among celiac cases, DQ2, DQ7 and DQ8 frequency in genotypes was 55, 24 and 22, respectively. Two cases (3.92%) were not HLA-DQ2/DQ8 carriers. As for FDRs, DQ8 was more frequent than among cases, with DQ2, DQ7 and DQ8 frequencies contributing 138, 82 and 82, respectively. Table 5 also shows that the most frequent genotype distribution was DQ2/DQ7 (fr 0.392 and 0.248 in cases and FDRs, respectively, *p* < 0.02). The second-most common genotype was DQ2/DQ8 (fr 0.236 in FDRs and 0.176 in cases, *p* < 0.05). DQ2/DQ2 was third in frequency, without differences between cases or FDRs. 3/48 cases. 

Among tTG positive FDRs, 57.4% carried DQ2 while 22.3% and 20.2% carried DQ7 and DQ8, respectively. This distribution was not different among tTG-negative FDRs. As for the four FDRs diagnosed CD, 2 carried DQ2/DQ2, 1 DQ2/DQ8 and 1 DQ2/DQ7.

## 4. Discussion 

### 4.1. Clinical Aspects

Results show that active search yielded 16.9% of tTG-positive FDRs, a figure that is in the higher range of values reported [22,32,33]. Overweight/obesity among FDRs was close to 50%, not different from the range currently described in our country’s general population, with no differences between symptomatic and subclinical individuals [15]; as expected, overweight/obesity was more frequent among tTG negative (57.2%) than tTG positive (35.7%, *p* < 0.01) FDRs; at the same time, under-nutrition was marginally present in all FDRs (Table 1). All this emphasizes the new scenario of CD. As described by other authors, symptoms in FDRs were less frequent (Table 2), mainly abdominal pain (28.9%) and constipation (18.1%) [22,27]. However, results showed some rather unexpected findings in this group; it was surprising that the majority of FDRs spontaneously declared they were asymptomatic, but the more detailed questioning revealed that 60.7% did experience some kind of symptoms; these were similar to those observed in celiac cases, and their frequency among tTG-positive FDRs was not different from that of tTG-negative FDRs (54.3%). It was not possible to clarify to what extent lower intensity of symptoms could be responsible for the lack of awareness of disease. It was unfortunate that only a few of the serologically positive FDRs accepted an endoscopic biopsy; this represents the main limitation to this study; reluctance to accept being investigated seems to be common among these individuals and has also been reported by other authors [34]; in the four newly diagnosed FDRs, DQ2 was present in all them.

Among celiac cases, at time of diagnosis, 35.7% of them required hospitalization, which we interpret as suggesting late consultation and/or diagnosis. This clearly draws attention to a public health problem, the need for promoting active search; at present, the large group of professionals responsible for public health systems does not apply it in several countries. As expected, the frequency of autoimmune disorders was found to be higher than in the general population [35,36]. Other conditions and diagnoses, such as anemia and short stature, were present both in celiac cases and FDRs. Time to consult since the appearance of symptoms was clearly longer among adult patients (data not shown), as also was the time to reach diagnosis; although the study was not designed to assess this issue, chart analysis suggested that at present, although diagnosis is indeed delayed, people in the community seem to be more aware of non-classical presentations of CD and spontaneously look for help earlier. The fact that about half of cases showed positive serologic studies at the time of our current assessment reveals poor adherence to treatment; unfortunately, this finding is not different from what we have found in previous evaluations [17].

### 4.2. Genetic Data

The results obtained in the celiac cases confirmed that DQ2 is the main allele associated with CD [14] and with more intense damage to the intestinal mucosa [37,38]. Although DQ2 exhibits the highest frequency in this study, it is clearly lower than reported (Table 4 and Table 5) [22]. DQ8 appears to be less frequent than in our own previous studies, sharing the second frequency with DQ7, a relevant finding considering similar recent publications in the South American region [18,20]. In relation to DQ8 findings, our present results agree with what we found in 2010 [19] and differ from older data obtained in 1999, which showed that DQ8 was more prevalent [17]. There is no clear explanation for the differences observed; the three groups assessed 15 years ago represented an unselected local population receiving medical care in the public health systems of Santiago. However, the protocols applied had different designs; the results of this study cannot clarify whether this may be responsible for the different results obtained. It is also worth mentioning that in the present study the frequency of cases not carrying HLA-DQ2 or HLA-DQ8 was 3.92%, a figure still higher than that reported in Europe, but somewhat lower than our own previous results that showed 7% [18].

In summary, results of this study contribute to improving global knowledge of CD, in this case in Chile as part of the southern cone of South America. tTG-positive FDRs were found at 16.9%, a figure in the higher range of those previously described, which contributes to characterizing this group; about half of FDRs were overweight/obese, emphasizing the new scenario of CD; and 60.7% reported symptoms which had not been investigated. This finding among FDRs, the long time elapsed between initiation of symptoms and diagnosis and the high percentage of cases meant to be treated but tTG-positive at the time of this study strongly stress the need for improving public health management of CD. Genetic analyses showed that HLA-DQ2 alleles were first in frequency (53.9%), but the figure found is lower than those reported elsewhere, with HLA-DQ7 and DQ8 making significant contributions, both as alleles and genotypes. These findings agree with recent communications originating from the same geographical region and suggest that, although CD may manifest clinically in ways similar to those described in other areas (mainly Europe and North America) in this region, it seems to have some genetic peculiarities that deserve further study.
